# Monitoring the Spatial Variation of Aerosol Optical Depth and Its Correlation with Land Use/Land Cover in Wuhan, China: A Perspective of Urban Planning

**DOI:** 10.3390/ijerph18031132

**Published:** 2021-01-28

**Authors:** Qijiao Xie, Qi Sun

**Affiliations:** 1 Faculty of Resources and Environmental Science, Hubei University, Wuhan 430062, China; sq1631@126.com; 2 Key Laboratory of Regional Development and Environmental Response (Hubei Province), Wuhan 430062, China

**Keywords:** AOD pollution, land use pattern, urbanization, spatial distribution, correlation analysis

## Abstract

Aerosols significantly affect environmental conditions, air quality, and public health locally, regionally, and globally. Examining the impact of land use/land cover (LULC) on aerosol optical depth (AOD) helps to understand how human activities influence air quality and develop suitable solutions. The Landsat 8 image and Moderate Resolution Imaging Spectroradiometer (MODIS) aerosol products in summer in 2018 were used in LULC classification and AOD retrieval in this study. Spatial statistics and correlation analysis about the relationship between LULC and AOD were performed to examine the impact of LULC on AOD in summer in Wuhan, China. Results indicate that the AOD distribution expressed an obvious “basin effect” in urban development areas: higher AOD values concentrated in water bodies with lower terrain, which were surrounded by the high buildings or mountains with lower AOD values. The AOD values were negatively correlated with the vegetated areas while positively correlated to water bodies and construction lands. The impact of LULC on AOD varied with different contexts in all cases, showing a “context effect”. The regression correlations among the normalized difference vegetation index (NDVI), normalized difference built-up index (NDBI), normalized difference water index (NDWI), and AOD in given landscape contexts were much stronger than those throughout the whole study area. These findings provide sound evidence for urban planning, land use management and air quality improvement.

## 1. Introduction

Aerosols are solid or liquid particles suspended in air with the size ranging from 0.001 to 10 μm. They are often observed as smoke, dust, fog, or haze, which significantly affect climatic change, environment condition, and air quality not only at a local scale, but also regional and global ones [1,2,3,4]. By absorbing or scattering incident electromagnetic radiation, aerosols have obvious radiative forcing effects on climate and environment [5,6] and also influence the radiative energy balance [7,8]. Fine particles and PM_2.5_, aerosols with aerodynamic diameters of less than 2.5 μm, can seriously endanger public health [9,10], due to the harm to the respiration and blood circulation system of inhabitants [11]. According to the World Health Organization, more than 7 million people in the world lose their lives every year due to PM_2.5_ pollution related diseases [12]. Aerosols are considered as one of the main air pollutants that affect air quality and jeopardize public health [13]. Aerosol Optical Depth (AOD), an important physical parameter, is defined as the integrated extinction coefficient over a vertical column of unit cross section [13,14]. It is closely related to the main pollutants such as PM_2.5_, PM_10_, NO_2_, SO_2_, and O_3_ [15,16] and widely used to indicate the atmospheric conditions, represent air pollution level, and describe climatic effects [13,17,18]. Thus, AOD and its driving factors have recently attracted a lot of attention [19,20,21].

Both natural sources and anthropogenic activities contribute to atmospheric aerosols [22]. Compared with natural aerosols such as sea spray, soil ash, volcanic dust, and biomass burning, those associated with human activities such as transportation, industrial emission, and household fuel combustion contribute more to aerosol pollution in and near urban areas [23,24]. Rapid urbanization and industrialization result in heavy urban traffics and increased industrial emissions. Concentrated and crowded urban residents increase household fuel consumption and domestic emission. In addition, urbanization tremendously alters urban surface structures with the original natural resources replaced by dense and high buildings [25]. Compact city layout reduces wind speed, affects aerosol diffusion and causes pollutant accumulation [26]. These consequently increase aerosol amount and concentration. However, the aerosol concentration is discontinuous and varies over the city due to the high spatial heterogeneity of underlying surfaces and pollution sources. Detecting the spatial complexity of AOD distribution and its response to urbanization helps to understand the impact of anthropogenic activities on aerosol pollution [27]. Previous studies on the relationship between urbanization and AOD spatial distribution confirmed that AOD distribution was significantly affected by local climate, population density, socio-economic conditions, and human activities [28,29,30]. Nevertheless, these variables lack of spatial accuracy and are difficult to be obtained directly. It limits the quantitative study of their influence on the spatial variation of AOD on a large scale.

Land use/land cover (LULC) structure or urban form is the direct result of the urbanization process [25,31]. The LULC pattern is closely related to the spatial distribution of population density, pollution sources, and meteorological conditions, which affects the AOD distribution and pattern. Both remote sensed technology and ground-based investigation are performed to investigate the relationship between LULC and AOD and AOD-related pollution [32,33,34,35]. The Moderate Resolution Imaging Spectroradiometer (MODIS) aerosol products provide spatially consistent data, which have been widely used in recent years [19,28,29,36,37,38]. For example, Guo et al. (2012) investigated the spatial distributions and temporal variations of AOD and their affecting factors in central China by using MODIS-retrieved aerosol level-2 C005 product [20]. Li et al. (2014) assessed the AOD distribution and its correlation with LULC and socio-economic factors over Guangdong Province from the aerosol product of MODIS at 10 km spatial resolution [29]. He et al. (2016) monitored the spatiotemporal variations in AOD and the impact factors over China based on the Level 2 aerosol products of MODIS with a 3 km spatial resolution [37]. Liu et al. (2020) examined the impact of LULC on AOD characteristics in central Asia based on the MODIS products with a spatial resolution of 10 km × 10 km [38]. Massive studies focused on the AOD properties and the impact factors over national and regional areas at a coarse spatial scale. However, both the formulation and implementation of the policies about LULC planning and air pollution management are more active within a city.

It is of great significance to investigate the impact of LULC on AOD and AOD-related pollution in different case cities. Extensive studies were performed based on the meteorological station records and in-site observations [13,39,40,41]. They were efficient in accurately measuring aerosols in different environmental contexts and offered detailed explanation for the spatial variation of aerosol size, composition, and concentration [42]. However, the collecting measurements are time-consuming and context-sensitive. The reliability and invalidity of the research results greatly depend on the number, frequency, and layout of the measurement points or meteorological stations [41]. The conclusions from the ground-based measurement provided sound support for policymaking in alleviating aerosol pollution on fine scales but lack of a synchronized view over the city [31,39]. They could not meet the demand for larger scale AOD assessment oriented to urban planning [43,44]. It is necessary to take the city as a complete system and monitor the spatial variation of AOD in it. Nevertheless, to date few studies on AOD distribution and its response to LULC were performed at the city scale [18,28,38].

This study, therefore, aims to detect the impact of LULC on AOD on the city scale based on the Landsat 8 image with the resolution of 30m and MODIS aerosol products with the resolution of 1km. The specific objectives are to (1) identify the LULC spatial pattern in Wuhan, China, (2) monitor the spatial variation of AOD values on the city scale, (3) quantitatively investigate the correlations between AOD and LULC-related variables, (4) detect how LULC influences the AOD variation in different contexts, (5) and discuss the implication of study results from the perspective of urban planning. The results can provide informative data for land use management and air quality improvement.

## 2. Materials and Methods

### 2.1. Study Area

**Figure 1:**
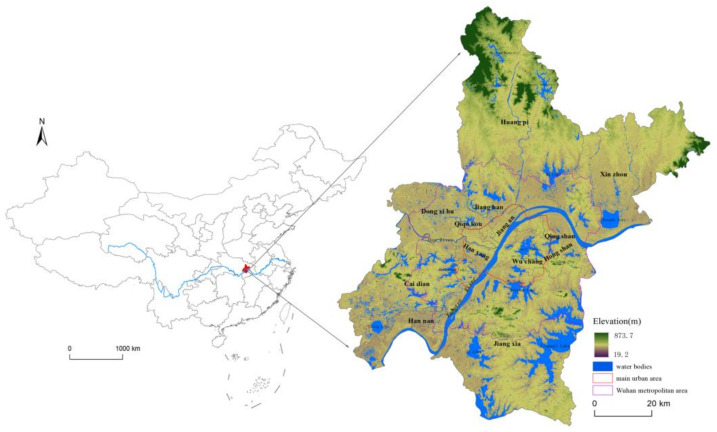
Location of Wuhan in China.

Wuhan (113°41′–115°05′ E, 29°58′–31°22′ N), the capital of Hubei Province, is located in the eastern margin of the Jianghan Plain and the southern foot of Dabie Mountain (Figure 1). The altitude of the study area ranges from 19.2 m to 873.7 m, with most regions being below 50 m. The city center is low and flat, surrounded by low mountains and hills. The plains and hills account for 81.9% and 18.1% of the total area, respectively. It has a subtropical monsoon climate with cold winter and hot summer. The annual average temperature varies between 15.8 °C to 17.5 °C, and the annual average precipitation ranges from 1150 mm to 1450 mm. The annual average wind speed is from 1.56 m/s to 2.73 m/s with the main directions of NNE and NE. In summer, the wind speed is relatively low and stable with the value of 1 m/s from the SW direction [45]. A crisscross water network was interwoven by different water bodies such as the Yangtze River, the Han River, Liangzi Lake, Tangxun Lake, and East Lake in Wuhan. Wuhan has a population of more than 11 million and a total area of over 8500 km^2^. It is composed of 13 administrative regions including Jiangan, Jianghan, Qiaokou, Hanyang, Wuchang, Qingshan, Hongshan, Dongxihu, Hannan, Caidian, Jiangxia, Huangpi, and Xinzhou districts.

With the sustaining and rapid development of urbanization and industrialization in recent years, Wuhan has been suffering from the serious air pollution and frequent extreme pollution events [46]. As recorded, during the four years from 2014 to 2017, about 89.4% of the daily air quality was assessed as polluted in Wuhan. The major pollutants are PM_2.5_, PM_10_, NO_2_, and O_3_, which are the main components or sources of aerosols [47]. Higher dustfall pollution level was detected in highly developed areas than that in clean environment due to the higher building density and lower wind velocity in urban areas [48]. The air pollution degree (with AOD as the indicator) significantly varies over space with higher spatial heterogeneity in Wuhan [49].

### 2.2. AOD Retrieval from MODIS Data

Multi-Angle Implementation of Atmospheric Correction (MAIAC) is a new algorithm for the aerosol retrievals and atmospheric correction of MODIS data [50,51]. The feasibility and applicability of the MAIAC AOD data have been verified by the ground-based aerosol records in different areas [52,53]. The MAIACAOD Level-2 gridded products (MCD19A2) provide informative data with a spatial resolution of 1 km and a temporal one of 1 day. They were widely used to monitor the spatiotemporal variation of AOD at city scales due to the high spatial resolution and high accuracy [18,28,51]. In this study, the MCD19A2 products in July, August, and September in 2018 were used to indicate the AOD variation in summer, which were obtained from the LAADS DAAC (data portal: https://ladsweb.modaps.eosdis.nasa.gov/search/). The MCD19A2 data offer daily-basis AOD values at the wavelength of 0.47 μm (blue band) and 0.55 μm (green band). In this study, AOD values at 0.55 μm were selected due to the relatively stable quality.

To alleviate the influence of meteorological conditions on AOD detection, those daily AOD products obtained in cloudy, rainy, or windy days were excluded. The remained 51 MCD19A2 products were first converted into Tiff format from HDF one using the MODIS Reprojection Tools (MRT), and then reprojected to the WGS_1984_UTM_Zone_50N coordinate system. Because the scale factor of the pixel for AOD_0.55 µm band is 0.001, it is necessary to reduce the pixel value by 1000 times before AOD statistics [54]. After basic processing, the daily MCD19A2 data were averaged to obtain the mean AOD values in summer in ArcGIS10.2 software (Esri, Redlands, California, USA). The pixels with missing AOD values due to the clouds and water vapor were filled in using the Kriging interpolation method [55]. The spatial distribution of AOD values in Wuhan was masked and cut out in ArcGIS according to the boundary of the study area.

### 2.3. LULC Classification Based on Landsat 8

#### 2.3.1. Supervised Classification Method

In this study, Landsat-8 OLI image at a 30 m resolution acquired on 15 September 2018 was used to map the LULC patterns. The data were downloaded from USGS (data portal: https://earthexplorer.usgs.gov/) with cloud coverage less than 10%, which met the study requirements. The data preprocessing of the remote sensing images, including geometric correction, radiometric calibration, atmospheric correction, image enhancement, image mosaic, and clipping was performed in ENVI5.3 (ITT VIS, Boulder, Colorado, USA) [56].

The supervised classification method was employed in ENVI5.3 to classify the image by a maximum likelihood algorithm. Considering the surface cover characteristics and AOD pollution properties, the land cover was divided into five major categories: (1) Farmland: refers to the agricultural land, including dry land, vegetable land, and paddy field but not orchards. (2) Forest: mainly includes large area of evergreen or deciduous arbors/shrubs with the canopy coverage more than 60%. (3) Greening land: refers to the vegetated areas except forest, including grassland, orchard, and sparse wood grassland. (4) Water bodies: includes all kinds of water cover such as rivers, lakes, wetlands, reservoirs, and fish ponds. (5) Construction land: mainly includes industrial areas, commercial areas, residential areas, transportation facilities, and other infrastructure land. For each LULC category, more than 250 samples were randomly selected to assess the classification accuracy based on the Google earth correction. The total accuracy and the kappa coefficient after classification were 87.64% and 0.83, respectively.

#### 2.3.2. Derivation of NDVI, NDBI and NDWI

Normalized Difference Vegetation Index (NDVI) is sensitive to vegetation and reflects the green biomass and plant growth status. It is usually used to indicate vegetation amount and factional vegetation cover. It can effectively eliminate or reduce the negative effects of topography, atmospheric radiation, cloud cover, and instrument calibration errors on vegetation quantification, so it is widely used in urban climate related research. NDVI integrates the visible and near-infrared reflectance spectral information, which can be obtained from

(1) $$NDVI=\left(\rho_{NIR}-\rho_{R}\right)/\left(\rho_{NIR}+\rho_{R}\right)$$

where $\rho_{R}$ is the reflectance value of red band (band 4) and $\rho_{NIR}$ is the reflectance value of near-infrared band (band 5) for Landsat 8 image. The NDVI values range from −1 to 1, where positive values indicate vegetation areas with larger values representing higher vegetation coverage, and negative values indicate non-vegetated surface features.

Normalized Difference Build-up Index (NDBI), an index sensitive to construction land, was widely used to indicate the development degree and density of built-up area. It can be used to differentiate the detailed LULC characteristics by identifying the appropriate threshold values according to the contexts of study area. Some previous studies suggested that NDBI was more suitable for quantitatively indicating LULC types and their spatial variation than NDVI [57]. The NDBI values were used to statistically model its relationship with AOD variation in this study. The formula is

(2) $$NDBI=\left(\rho_{MIR}-\rho_{NIR}\right)/\left(\rho_{MIR}+\rho_{NIR}\right)$$

where $\rho_{MIR}$ and $\rho_{NIR}$ are the reflectance values of the mid-infrared band and near-infrared one, respectively. The values range from −1 to 1 with higher values indicating higher building density.

Another index used in this study is Normalized Difference Water Index (NDWI), which could effectively express the water information and can be derived by

(3) $$NDWI=\left(\rho_{G}-\rho_{NIR}\right)/\left(\rho_{G}+\rho_{NIR}\right)$$

where $\rho_{G}$ is the reflectance value of green band (band 3 for Landsat8 images).

### 2.4. Data Analysis

In order to investigate the spatial relationship between AOD distribution and LULC pattern at the same resolution, the AOD data and LULC-related indicators were resampled into the grids sized in 1 km × 1 km in ArcGIS10.2 software using the Fishnet tool. A total of 9008 grids were generated in the study area. For each grid, the average values of AOD, NDVI, NDBI, NDWI, and the area proportion of different LULC types such as construction land (PerCon.), farmland (PerFarm), forest (PerForest), greening land (PerGreen), and water body (PerWater) were calculated. The Pearson correlation analysis between AOD and the LULC-related indicators was performed in SPSS19.0 (IBM, Armonk, NY, USA). In addition, the preliminary quantitative relationship between AOD and the indexes was analyzed based on scatter diagram.

## 3. Results

### 3.1. AOD Spatial Distribution

**Figure 2:**
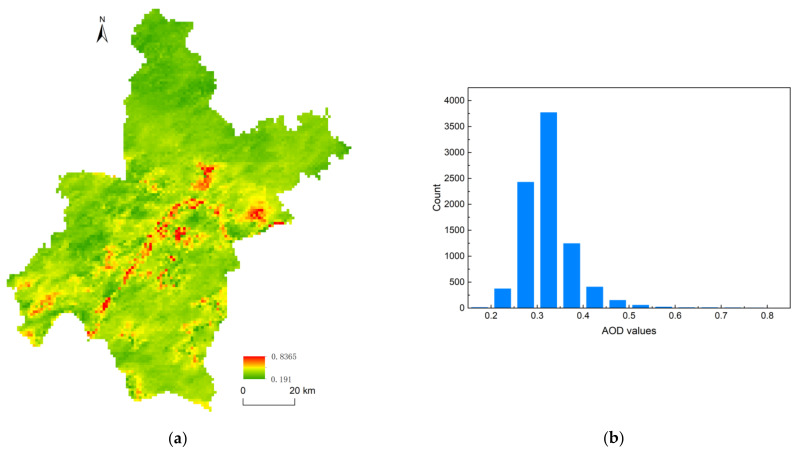
The distribution maps of AOD (Aerosol Optical Depth) values in Wuhan: (**a**) spatial distribution and (**b**) histogram distribution.

Figure 2 shows the spatial pattern and histogram distribution of AOD values. As Figure 2a shown, the AOD pollution was not serious in Wuhan during the study period. The most frequent AOD values were mainly concentrated from 0.2–0.5, covering about 98.6% of the study area (Figure 2b). Only 1.31% of the Wuhan administrative region was covered by AOD value higher than 0.5. Nevertheless, the spatial distribution of AOD values was not stable and continuous and there were significant spatial differences in varied regions. The AOD values ranged from 0.191 to 0.837 over the study area with the average one of 0.32 (standard deviation of 0.06). The highest value occurred in the Yangtze River, a pixel located in the city core. The lowest one appeared in a water pixel in the Xiajiasi Reservoir in the north of Wuhan. Higher AOD values were mainly distributed in the middle and south Wuhan, especially in the water bodies in highly developed areas. Lower ones were almost concentrated in the north and northeast Wuhan, where natural mountains with high vegetation coverage prevailed. Generally, the AOD variation over study area was related to the LULC pattern.

### 3.2. Land Use Pattern

**Figure 3:**
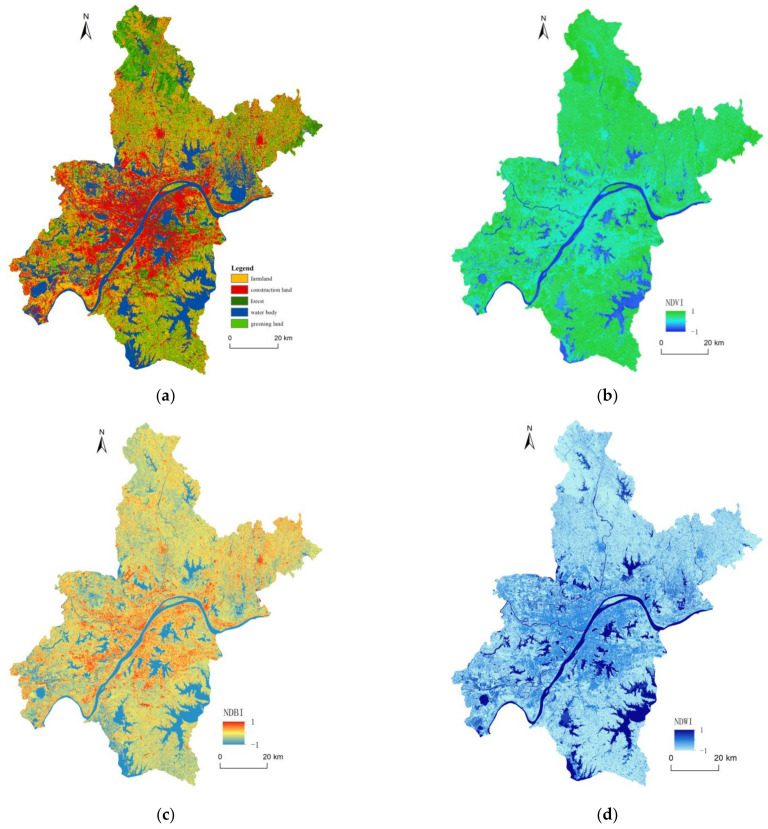
Spatial distribution maps of (**a**) LULC (Land use/Land cover), (**b**) NDVI (Normalized Difference Vegetation Index), (**c**) NDBI (Normalized Difference Built-up Index) and (**d**) NDWI (Normalized Difference Water Index) values.

Land use is the closest link between human beings and nature. LULC patterns record the way and result of human action on nature and the environment. Figure 3a shows the spatial distribution of LULC pattern in Wuhan in 2018. Table 1 displays the area statistics of different LULC categories. The results indicated that the farmland areas were about 2514.2 km^2^, accounting for the largest proportion of 29.3%. They were mainly distributed in the suburbs such as Huangpi, Jiangxia, Xinzhou, and Hannan districts, around the city core. The construction lands were mostly concentrated in the city center or sprawled along the main roads in suburbs, covering approximately one-quarter of the whole study area (2022.3 km^2^). Together with the urban lakes such as East Lake, South Lake, Shahu Lake, and Liangzi Lake, the Yangtze River and Han River pass through Wuhan City. With the covering area of 1706.2 km^2^, water bodies accounted for a large percentage (19.9%) in the study area. Vegetated areas, namely, forests and greening lands, were staggered with the other LULC types throughout the whole study area. They had a total area of 2336.4 km^2^ (27.2%), among which forests covered 533.3 km^2^. The concentrated forest was detected mainly in the north and northeast in Wuhan.

Figure 3b illustrates the spatial distribution map of NDVI values in Wuhan in 2018. Higher NDVI values typically represent larger vegetation amount and greater vegetation coverage. The dark blue areas with negative NDVI values were almost restricted to water bodies. The light blue areas covered by medium NDVI values were detected to well correspond to the construction land both in urban built-up areas and rural developed areas. As expected, higher NDVI values were found in the surrounding suburb areas where plantation or native forests dominate. The NDBI distribution map is illustrated in Figure 3c, which was obtained from Landsat 8 image. It provided continuous variation trends with the increasing gradation in positive values from undeveloped area to slightly developed area to moderately developed area then to highly developed area. The areas covered by negative values expressed good spatial consistency with the distribution of water bodies. Figure 3d drafts the NDWI distribution map with the values ranging from −1 to 1. It expressed continuous variation of water properties at pixel level and provided specific data for the impact analysis of water body on AOD variation.

### 3.3. Impact of LULC on AOD Spatial Distribution

#### 3.3.1. Impact of LULC on AOD Variation

To compare the AOD variation within and among different LULC types, the minimum, maximum, average, and standard deviation (SD) values of AOD in different LULC categories were counted, as shown in Table 2. The areas covered by Water body exhibited the highest mean AOD value of 0.358, followed by Construction land (0.326), Farmland (0.317), Greening land (0.311), and Forest (0.294). Although the reservoirs in the northern suburbs had relatively low AOD values, most of water bodies (especially in urban areas) had significantly higher ones (Figure 2a), which led to the highest mean AOD value in water bodies. It was noticeable that both the minimum value and maximum one were detected in water bodies, which had the widest range of AOD values. Significant variation of aerosols existed within Water body category with the SD value of 0.081, the largest in all LULC types. Generally, vegetated areas covered by Forest, Greening land, and Farmland had relatively lower mean AOD values. Among them, Forest had the lowest mean and SD values of AOD, indicating that densely vegetated areas provided a cleaner environment.

To better understand the relationship between LULC and AOD values, several indexes associated with LULC were selected and their correlations with AOD were assessed in this study. The correlation coefficients between AOD values and LULC-related variables were calculated and shown in Table 3. Results indicated that all the selected indexes except NDBI were closely correlated with AODs at the significance level of 0.01. As expected, the indexes associated with built-up areas such as PerCon. and NDBI, were both positively correlated with AOD. However, the correlation coefficients were only 0.025 and 0.045, respectively. Significant negative relationships existed between AOD values and all the indicators related to vegetation coverage, namely, NDVI, PerForest, PerGreen, and PerFarm. Vegetation was confirmed to have a positive purification effect on aerosols due to the adsorbing and removing capacity of green leaves. It is noteworthy that there were significant positive correlations between NDWI, PerWater, and AOD values, with relatively higher coefficients of 0.423 and 0.425, respectively.

#### 3.3.2. Relationships between AODs and the Values of NDVI, NDBI and NDWI

The above results indicated that AOD values were significantly affected by LULC types. To further investigate the impact of LULC on AOD distribution, the scatter plots with density for the relationships between NDVI, NDBI, NDWI, and AOD were illustrated. In addition, a method of zonal analysis was carried out to evaluate the mean AOD values at each 0.01 increment of NDVI, NDBI, and NDBI from −1 to 1.

**Figure 4:**
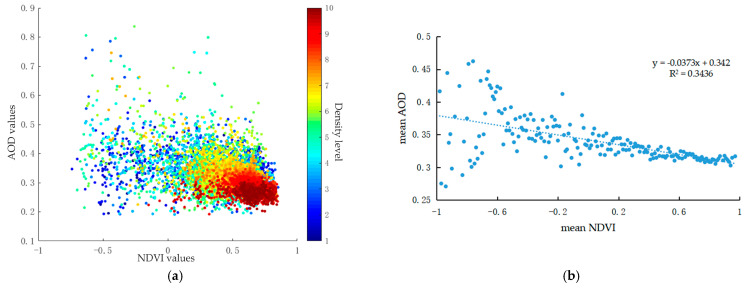
Relationship between AOD (Aerosol Optical Depth) and NDVI (Normalized Difference Vegetation Index) values: (**a**) scatterplots and (**b**) regression equation.

Figure 4 displays the quantitative relationship between AOD and NDVI values. As shown in Figure 4a, though negative correlation was detected between AOD and NDVI, the relationship in scatterplots with density between AOD and NDVI was weak and nonlinear. This means that no strong spatial relationship exists between vegetation and aerosols. This was partly attributed to the wide and fragmented distribution of the vegetated areas on the city scale. The scatter plots were mainly clustered in the NDVI values of 0–1, where the densely vegetated areas were dominant and concentrated. Figure 4b drafts the regression equation between the mean AOD and mean NDVI values, expressing the changing trend of AOD as the mean NDVI varied. The mean AOD was found to be correlated to the mean NDVI with the R^2^ value of 0.3436, suggesting that aerosol concentration was influenced by vegetation coverage. The negative relationship indicated that AOD decreased as NDVI increased. Increasing vegetation coverage is of great significance to alleviate air pollution.

**Figure 5:**
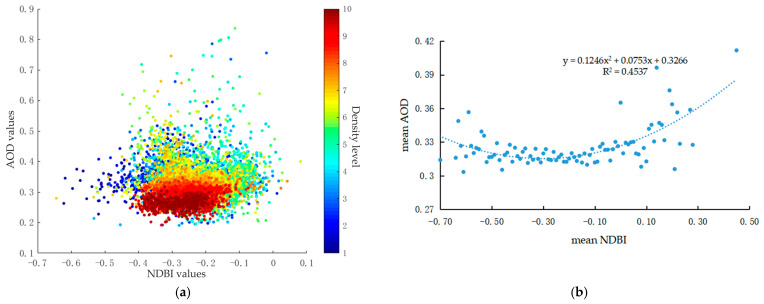
Relationship between AOD (Aerosol Optical Depth) and NDBI (Normalized Difference Build-up Index) values: (**a**) scatterplots and (**b**) regression equation.

Figure 5 indicates the quantitative relationship between AOD and NDBI values. For the whole study area, natural areas covered the majority and construction land accounted for about 25% (shown in Table 1). The built-up areas were fragmented by water bodies and the vegetated areas, which resulted in the densely distributed cluster with the NDBI from −0.5 to 0.1. The weak relationship in scatterplots with density between AOD and NDBI, shown in Figure 5a, meant that NDBI alone might not efficiently explain the aerosol spatial variation. The association between mean AOD and mean NDBI (Figure 5b) was nonlinear and relatively weak with the R^2^ value of 0.4537. The regression curve of these two elements first showed a negative correlation and then a positive one. In the areas with NDBI less than 0, the mean AOD values decreased with the mean NDBI increased. The effect of built-up area on aerosol concentration was significantly influenced by other factors in natural context, which weakened their correlation.

**Figure 6:**
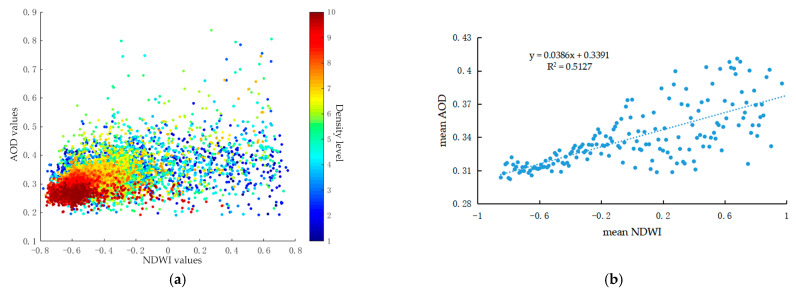
Relationship between AOD (Aerosol Optical Depth) and NDWI (Normalized Difference Water Index) values: (**a**) scatterplots and (**b**) regression equation.

Figure 6 demonstrates the quantitative relationship between AOD and NDWI values. Due to the limited area and sparsely distributed pattern of water bodies (Table 1 and Figure 2), the majority of pixels were concentrated in the range with the NDWI values of −0.8 to −0.2. The scatterplots with density between AOD and NDWI (Figure 6a) exhibited a relatively weak spatial correlation between aerosol amount and water body distribution on city scale. However, AOD values were more consistent with NDWI in urbanized areas than in suburbs, which was also supported by the AOD distribution characteristics (Figure 2a) and NDWI pattern (Figure 3d). As Figure 6b shows, the regression equation between the mean AOD and mean NDWI first showed strong relation and then weak one. With the mean NDWI increasing, the correlation between mean NDWI and mean AOD was affected by other factors more frequently. The positive correlation between these two elements meant that increasing the area of water bodies could promote the aerosol deposition.

## 4. Discussion

### 4.1. Basin Effect of AOD Distribution in Urbanized Area

When masking the AOD pattern (Figure 2a) over the LULC map (Figure 3a), we found that the aerosol distribution did not always correspond to land use types. However, relatively higher AOD values tended to be concentrated in the water bodies in the urban development areas. As shown in Figure 1, the urban development area of Wuhan was mainly located at flatland with less internal variation in altitude. Surrounded by the dense and high buildings, water bodies in urbanized areas formed vivid “basin” shapes. Urban development increased the requirement of infrastructure construction, led to frequent human activities and consequently resulted in increasing emission of anthropogenic aerosol pollutants in urban areas [27,28]. On one hand, the surrounding buildings disrupted the horizontal dispersion of the aerosol pollutants, forcing aerosols to concentrate in open areas with lower terrain [58]. On the other hand, higher relative humidity over the water bodies increases the particle hygroscopic and volume of fine particles, which raises the aerosol concentration [59]. Thus, water bodies within or near to the urban development areas, such as the Yangtze River, Han River, Liangzi Lake, and Wu Lake, were covered by the highest AOD values. In contrast, the other LULC areas such as construction land, greening land, and farmland were generally captured by relatively low AOD values (Figure 2b). The AOD spatial distribution expressed an obvious “basin effect” [60,61]: high AOD values primarily concentrated in water bodies with lower terrain, and low AOD ones generally distributed in the surrounding areas with high buildings or mountains.

Noticeably, there was a significant difference in AOD values between urban and suburb water bodies. In our study, the “basin effect” was only detected in urbanized areas. The water bodies in the city core had higher AOD concentrations. However, those in the north and northeast of Wuhan, such as the Meidian Reservoir, Yuanjisi Reservoir, and Xiajiasi Reservoir, were covered by lower AOD values. There was no difference in AOD between the reservoirs and the surrounding forests (Figure 2b). It was partly attributed to the clean environment in the natural mountains, which was less disturbed by human beings and far away from the anthropogenic pollution sources. Lower aerosol concentrations were diluted or absorbed by the natural vegetation.

### 4.2. “Context Effect” of LULC on AOD Variation

The mean AOD values and their variation range over different LULC categories showed discernible differences (Table 2) and the LULC-related indicators were all significantly correlated with AOD (Table 3). LULC and its structure did contribute to AOD variation on different scales [13,38,42]. Nevertheless, the correlation coefficients between NDVI, NDBI, NDWI, and AOD were not high enough, with the values of 0.3436, 0.4537, and 0.5127, respectively (Figure 4b, Figure 5b, and Figure 6b). It meant that there was no distinct and continuous relationship between AOD and LULC on the city scale [37], and the indexes of NDVI, NDBI, and NDWI alone could not effectively explain the AOD variation over the study area.

**Figure A1:**
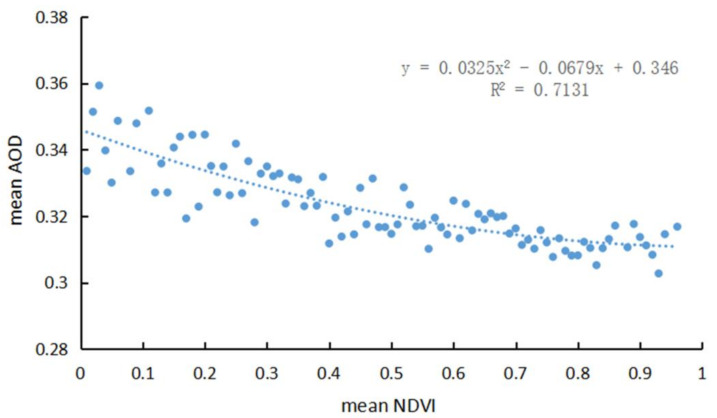
Relationship between NDVI (Normalized Difference Vegetation Index) and associated AOD (Aerosol Optical Depth) values in the NDVI range of 0 to 1.

It is remarkable that the regression correlations between NDVI, NDBI, NDWI, and AOD became much stronger when appropriate landscape context was identified or limited. Appendix A Figure A1 displays the relationship between NDVI and the associated AOD values during the range of NDVI from 0 to 1 (defined as vegetated area). The correlation coefficient was 0.7131, much higher than that over the study area (0.3436). Increasing vegetation coverage in vegetated or natural context was more efficient in removing aerosols than in the whole study areas. The removal and purification effect of vegetation on aerosols in artificial environment was less effective because it was significantly influenced by other factors such as ventilation, topography, and pollutant concentration [29,47].

**Figure A2:**
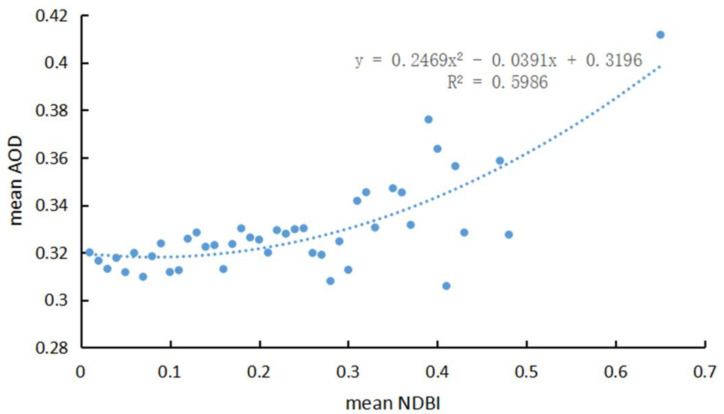
Relationship between NDBI (Normalized Difference Built-up Index) and associated AOD (Aerosol Optical Depth) values in the NDBI range of 0 to 1.

**Figure A3:**
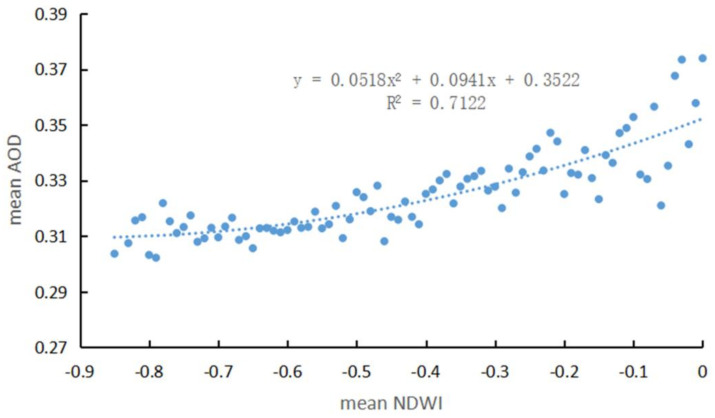
Relationship between NDWI (Normalized Difference Water Index) and associated AOD (Aerosol Optical Depth) values in the NDWI range of −1 to 0.

In general, the effect of NDVI and vegetation on AOD varied with different landscape contexts [37,47]. Similar conclusions can be drawn in the other two cases. As shown in Appendix A Figure A2 and Figure A3, the relationships between the mean NDBI, NDWI, and AOD were much stronger in the range of NDBI from 0 to 1 (R^2^ of 0.5986) and NDWI from −1 to 0 (R^2^ of 0.7122) than those throughout the study area. The impact of LULC on AOD was significantly influenced by the context characteristics, showing a “context effect”. It suggested that appropriate indexes should be chosen to indicate LULC according to varied landscape contexts, so as to better explain AOD variation.

### 4.3. Enlightenment on Urban Planning to Alleviate AOD Pollution

Our study confirmed that land use and its distribution significantly influenced AOD variation over space at a city scale (Figure 2 and Figure 3 and Table 3). Optimization of city layout and rationalization of land use allocation can play a vital role in mitigating air pollution and improving air quality [43,62,63]. In recent years, more and more researchers have been suggesting that climatic knowledge should be incorporated into urban planning practice to adapt to climate change and deal with negative environmental problems at the city scale [31,33,36,44].

Vegetation had the purification capacity of AOD pollution with the significant correlation between AODs and vegetation-related variables (Table 3). However, the influence of vegetation coverage in urban areas was more complex than that in other areas (Figure 3b and Figure 4b). Vegetation plays an important role in improving air quality by affecting both the deposition and dispersion of pollutants [47,62]. Green leaves of plants can absorb or remove air pollutants from the surrounding areas, and the rough leaf surface and complex canopy properties can increase the deposition velocity of pollutants and clear the environment [64,65]. Urban green areas with enough open spaces and sparse tree canopy form a ventilation channel and allow the air pollutants to pass through, which increases the pollutant dispersion and reduces the pollutant concentration in urbanized areas [62,66]. In contrast, densely planted large trees with crowed branches and canopies in urban areas are natural barriers between pollution sources and the surroundings, which may reduce the dispersion and increase the local pollution levels [67]. Thus, in terms of the alleviation of AOD pollution, urban vegetation should be dense enough to provide deposition area and porous enough to increase dispersion [67,68].

High concentration of AOD was mostly concentrated in large water bodies in and near the city core (Figure 2). Aerosol particles are blocked in urban areas by high-rise buildings, and they can be deposited when they pass over the open spaces [67]. The probability of pollutant deposition may be increased over water bodies due to the higher vapor content and the large open surface area [36]. Water bodies in urban areas then become the important sinks of air pollutants, which changes the distribution of pollutants within the city and reduces the pollution concentration in human living space indirectly [63,67]. Protecting large water bodies or connecting small-sized ones to form a water network are necessary for air quality improvement in urbanized areas. However, the transportation of the pollutants is closely related to meteorological conditions [4,20,30]. When wind speed and direction significantly change, the AOD pollutants gathered in water bodies may become pollution source and be blown back to residents’ living space. Urban blue-green infrastructure development mainly alters the internal deposition and dispersion of AOD pollutants, instead of the total pollutant amount in urban areas. Comparatively, suburb forests are more efficient in air pollution removal due to the large amount of green biomass (Figure 2 and Appendix A Figure A1). They can be used as natural sinks of urban AOD pollution. Connecting urban open spaces with suburban natural resources to build urban–rural ventilation corridor and ecological network is an effective measure to accelerate AOD dispersion and alleviate urban pollution.

### 4.4. Limitations and Research Prospects

Atmospheric aerosol concentration was mainly attributed to the anthropogenic emission of air pollutants in urban areas [40]. Investigating the impact of LULC on aerosols helps urban ecologists, climatologists, and planners to understand the interaction between human activities and environmental quality [38,69,70]. The results provided sound evidence for land use planning and air quality management. However, there still existed some limitations in this study.

First, in our study, water bodies, especially those in urbanized areas, were observed to be covered by high AOD values (Figure 2). Positively significant correlation was detected between AOD and water body (Table 3 and Figure 6). This finding was consistent with the results from Luo et al. (2001) [61] and Che et al. (2009) [59], but contrary to those from Zhu et al. (2019) [56] and Halim et al. (2020) [34]. Rich water resources and the crisscross water network in our study area were partly responsible for it. However, how water bodies influence the aerosol concentration and AOD distribution in detail is still unknown, which needs further investigation.

Second, vegetation affects aerosol and its distribution both directly and indirectly, by absorbing or depositing pollutants and influencing pollutant diffusion [71]. The purification capacity of vegetation varied with the biomass, vegetation coverage, vertical structures, and plant characteristics [72,73]. It is necessary to distinguish different vegetation types when observing the effect of vegetation on AOD [47,72]. Taking this into account, forest was discussed separately from vegetation in this study. Difference was found between forests and the other vegetated areas (Table 2 and Table 3), which supported the necessity. Further studies may focus on the detailed classification in vegetation and its impact on aerosols and air pollution.

Finally, our study focused on the impact of LULC on AOD based on the characteristics of different LULC underlying types without considering the interaction effects of LULC-related variables on AODs. While correlations were generally detected among all involved LULC-related variables when considering their impact on AOD variation, shown in Appendix A Table A1. In addition, AOD variation over spaces was also related to the specific land use properties. For example, pollutant amounts and types significantly varied in industrial land, commercial land, residential land, and traffic land, which then had different contributions to aerosols and their distribution [74]. Yet, most present studies grouped the above land types into built-up areas when discussing their effect on air pollution [33,34,38,75]. These need to be further improved in the future research.

## 5. Conclusions

This study focused on the impact of LULC on AOD in Wuhan, China. It provides a new perspective to understand how aerosol variation responds to land use layout at a city scale. The MODIS aerosol products with a spatial resolution of 1km×1km were used to map the AOD distribution. The Landsat 8 image with a spatial resolution of 30m×30m was used to obtain the LULC classification. Based on these databases, variability in AOD values over different LULC categories was counted, and correlation analysis between AOD and LULC-related variables was performed. Generally, the AOD distribution expressed an obvious “basin effect”: high AOD values primarily concentrated in water bodies with lower terrain, and low AOD ones usually distributed in the surrounding areas with high buildings or mountains. Among different LULC types, water bodies exhibited the highest mean AOD value (0.358), followed by Construction land (0.326), Farmland (0.317), Greening land (0.311), and Forest (0.294). AOD was confirmed to be closely correlated with NDBI and the other variables at the significance levels of 0.01 and 0.05, respectively. The positive correlation between AOD and built-up areas meant that urban development increased the aerosols and air pollution on a city scale. AOD was negatively correlated to vegetated areas such as forest, farmland, and greening land. Increasing vegetation coverage helps to promote the purification efficiency in aerosols. Our results confirmed that LULC and its structure did contribute to aerosols and its variation. However, the impact of LULC on AOD varied with different contexts in all cases, showing a “context effect”. For example, the “basin effect” was only detected in water bodies in urbanized areas. The purification effect of vegetation on aerosols was more efficient in vegetated areas than in the whole study area. Building urban–rural ventilation corridor and systemic ecological network can be effective in accelerating AOD dispersion and alleviating urban pollution. These findings could help urban planners and managers to develop appropriate strategies in urban planning and land use management.

## Figures and Tables

**Table 1:** Area statistics of different LULC (Land use/Land cover) categories in 2018 in Wuhan.

LULC Type	Area (km^2^)	Proportion (%)
Water body	1706.2	19.9%
Farmland	2514.2	29.3%
Greening land	1803.1	21.0%
Forest	533.3	6.2%
Construction land	2022.3	23.6%
Total	8579.1	100%

**Table 2:** Statistics of mean AOD (Aerosol Optical Depth) values in different LULC (Land use/Land cover) types in Wuhan.

LULC Type	Min	Max	Mean AOD	SD
Water body	0.191	0.837	0.358	0.081
Farmland	0.196	0.748	0.317	0.042
Greening land	0.196	0.537	0.311	0.040
Forest	0.202	0.474	0.294	0.051
Construction land	0.202	0.799	0.326	0.044

**Table 3:** Correlation coefficients between AOD (Aerosol Optical Depth) and LULC (Land use/Land cover) -related variables.

	NDVI	NDBI	NDWI	PerCon.	PerForest	PerGreen	PerFarm	PerWater
AOD	−0.422 **	0.025 *	0.423 **	0.045 **	−0.314 **	−0.298 **	−0.210 **	0.425 **

Note: ** Correlation is significant at the 0.01 level 2-tailed; * Correlation is significant at the 0.05 level 2-tailed. NDVI, normalized difference vegetation index; NDBI, normalized difference built-up index; NDWI, normalized difference water index; PerCon., area proportion of construction land; PerFarm, area proportion of farmland; PerForest, area proportion of forest; PerGreen, area proportion of greening land; PerWater, area proportion of water body.

## Data Availability

Data sharing not applicable.

## Appendix A

**Table A1:** Pearson correlations among the AOD values and the LULC-related variables.

	AOD	NDVI	NDBI	NDWI	PerCon.	PerForest.	PerGreen.	PerFarm.	PerWater.
AOD	1								
NDVI	−0.422 **	1							
NDBI	0.025 *	−0.283 **	1						
NDWI	0.423 **	−0.987 **	0.147 **	1					
PerCon.	0.045 **	−0.210 **	0.820 **	−0.243 **	1				
PerForest.	−0.314 **	0.346 **	−0.260 **	−0.311 **	−0.256 **	1			
PerGreen.	−0.298 **	0.682 **	−0.467 **	−0.644 **	−0.443 **	0.365 **	1		
PerFarm.	−0.210 **	0.550 **	−0.153 **	−0.546 **	−0.236 **	−0.240 **	0.193 **	1	
PerWater.	0.425 **	−0.769 **	−0.243 **	0.839 **	−0.301 **	−0.248 **	−0.503 **	−0.499 **	1

Note: ** Correlation is significant at the 0.01 level 2-tailed; * Correlation is significant at the 0.05 level 2-tailed. NDVI, normalized difference vegetation index; NDBI, normalized difference built-up index; NDWI, normalized difference water index; PerCon., area proportion of construction land; PerFarm, area proportion of farmland; PerForest, area proportion of forest; PerGreen, area proportion of greening land; PerWater, area proportion of water body.
